# A Concept for Extending the Applicability of Constraint-Induced Movement Therapy through Motor Cortex Activity Feedback Using a Neural Prosthesis

**DOI:** 10.1155/2007/51363

**Published:** 2007-09-23

**Authors:** Tomas E. Ward, Christopher J. Soraghan, Fiachra Matthews, Charles Markham

**Affiliations:** ^1^Department of Electronic Engineering, National University of Ireland, Maynooth, County Kildare, Ireland; ^2^Department of Computer Science and Department of Experimental Physics, National University of Ireland, Maynooth, County Kildare, Ireland; ^3^Hamilton Institute, National University of Ireland, Maynooth, County Kildare, Ireland; ^4^Department of Computer Science, National University of Ireland, Maynooth, County Kildare, Ireland

## Abstract

This paper describes a concept for the extension of constraint-induced movement therapy (CIMT) through the use of feedback of primary motor cortex activity. CIMT requires residual movement to act as a source of feedback to the patient, thus preventing its application to those with no perceptible movement. It is proposed in this paper that it is possible to provide feedback of the motor cortex effort to the patient by measurement with near infrared spectroscopy (NIRS). Significant changes in such effort may be used to drive rehabilitative robotic actuators, for example. This may provide a possible avenue for extending CIMT to patients hitherto excluded as a result of severity of condition. In support of such a paradigm, this paper details the current status of CIMT and related attempts to extend rehabilitation therapy through the application of technology. An introduction to the relevant haemodynamics is given including a description of the basic technology behind a suitable NIRS system. An illustration of the proposed therapy is described using a simple NIRS system driving a robotic arm during simple upper-limb unilateral isometric contraction exercises with healthy subjects.

## 1. INTRODUCTION

Strokes are characterised by an acute, nonconvulsive loss of neurological function as a result of an ischemic or hemorrhagic intracranial vascular event [1]. Worldwide, there are over 20 million cases of stroke each year [2] and of these approximately 75% are nonfatal with survivors left with a spectrum of poststroke disabilities ranging from mild numbness to severe motor and cognitive impairments. The dysfunction introduced depends on the site and extent of the infarction. Immediately following stroke, there is generally some degree of spontaneous recovery where some lost function is restored as a result of collateral circulation, reduction in inflammation, and haematoma compression among other factors. However, there are in nearly all cases significant residual neurological impairment, which if left untreated will result in severe degradation in life quality for the survivor. It is not surprising then that stroke is the leading cause of physical disability in Europe and the United States [3], and with 75% of stroke survivors suffering syndromes severe enough to affect their employability, the economic cost of the cerbrovascular disease stretches far beyond the immediate medical one. Poststroke rehabilitation is therefore critical to restore as much function as possible for the patient. This takes the form of neuro-rehabilitation, an interdisciplinary branch of clinical and medical science; the purpose of which is to restore neurological function and quality of life to people following disease or injury of the nervous system. Neurorehabilitation science draws on many techniques and therapies but the cornerstone of most treatments lies in physical therapy as most strokes seem to result in some form of hemiparesis or hemiplegia usually contralateral to the site of the stroke. The basic tenet of physical therapy or motor rehabilitation as it is often termed is that repetitive practise of proscribed movement can have a highly significant effect on rehabilitation outcome [4].

Currently, a particular type of motor rehabilitation termed constraint-induced movement therapy (CIMT) [5, 6] has been shown to be highly effective for use in hemiplegic stroke rehabilitation [7]. CIMT requires the subject have the unaffected limb constrained while they are encouraged actively to use the affected limb over long periods. With large periods of practise, the weakened side is strengthened significantly—possibly as a result of cortical reorganisation and changes in motor cortex excitability [8]. It appears that this method has statistically significant outcome improvements over equal intensity approaches [9, 10] and is currently the focus of concerted refinement and development to expand its theoretical basis and extend the application scope [11].

The flurry of research around this technique has had an impact on rehabilitation engineering where over the past few years the therapy has been augmented by robot-assisted training [12]. Such techniques extend CIMT to patients who have such severe disability that they are unable to engage in unassisted movement. This concept is underpinned by studies which have shown that the benefits of CIMT could be extended to such patients through the application of external forces applied to the limb [13] or functional electrical stimulation [14, 15]. The philosophy behind the approach envisaged in this paper is that perhaps the CIMT process can be enhanced further for this group if some measure of attempted activity in the motor regions could be presented either as a direct form of biofeedback or harnessed as a trigger to induce robotic–assisted movement or FES. Functional magnetic resonance imaging (fMRI) studies have shown that increases in bilateral cortical activation [16] are exhibited during CIMT; therefore appropriate feedback could be established using a brain-machine interface driven by signals derived directly from the motor cortical areas —a neural or more specifically, neurocortical prosthesis. It has been widely reported that NIRS systems are capable of detecting haemodynamic changes associated with motor movement both imagined and executed [17–19]; therefore an ideal neural prosthesis for this application is a NIRS-based brain computer interface (NIRS-BCI). The successful evoking of cortical NIRS responses could serve as the triggering event for biofeedback. NIRS has the additional benefit of showing oxyhaemoglobin (HbO) level changes as well as blood volume and deoxyhaemoglobin (Hb) changes which are the reported etiology of the MRI signal. The idea of extending the biofeedback loop directly to the motor areas responsible for movement is novel in this case of CIMT and the technique sits well with current opinions in neurorehabilitation which advocate enhanced motor learning techniques [18, 20]. The remainder of this paper is as follows. Section 2 begins with a description of the rehabilitation context for stroke survivors including a short presentation of the CIMT model. In addition, some background to BCI and robotics in a neurorehabilitation context is given including the relevant physiological measurement modality of NIRS. Section 3 comprises a technical illustration of the proposed concept to facilitate an appreciation for obstacles and issues facing practical embodiments of the idea. Section 4 discusses implications and prospects before a short summary is given.

## 2. BACKGROUND AND RELATEDWORK

Stroke rehabilitation therapies have until recently been characterised by empirically derived approaches rather than on the basis of scientifically derived theories. Traditional practises have a compensatory philosophy where targeted muscular and action accommodation techniques serve to circumnavigate impaired function. Lack of standards, poor validation, poor evaluation, and above all the lack of a neuroscientific basis has meant that neurorehabilitation clinical practises have languished outside the realm of evidence-based medicine. The advent of CIMT changed this perception and revolutionised rehabilitation medicine. CIMT is derived from a rigorously constructed conceptual framework which has its origins in the theory of learned non-use—an explanation for certain neurocortical and behavioural phenomena evident in monkey models of neurological dysfunction. [21]. In such models, the consequence of the paretic limb, for example, is the onset of a neuroplastic process in which the motor circuits undergo alteration which has degenerative impacts for the affected limb. CIMT aims to undo the learned nonuse through constraining the unaffected limb and forced repetitive training—a practise somewhat analogous to the use of the eye patch in amblyopia or lazy eye. With its efficacy confirmed during the largest ever controlled trial in neurological rehabilitation [10], the continued clinical practise of this therapy and its further refinement and extension are assured.

The rigorous psychological and neurological basis underlying CIMT makes it very amenable to integration with assistive technologies which yield quantitative measures and assessment criteria. The development of robotic actuators in tandem with neural prosthetic devices for such rehabilitation procedures is a natural development for future CIMT variants. The recent literature exhibits a growing and versatile range of potential systems that may be effective in application with CIMT.

### 2.1. Robot-assisted neurorehabilitation

The use of robotic systems as aids in neurorehabilitation is not new with systems such as MIT-MANUS [22] demonstrating the efficacy of the technique almost ten years ago. Their application to neurorehabilitation is quite natural as it is well known that intensive goal-directed movement repetition facilitates improved recovery outcome following stroke [23, 24] and as robots can engage in repetitive tasks consistently and unobtrusively, they are excellent deliverers of rehabilitation therapy. Clinical effectiveness has been reported in several studies [25–27] and it seems that these rehabilitative devices will be incorporated into standard clinical practise in the near future. Comprehensive reviews of such devices and their efficacy can be found in the literature [28, 29]. Such systems also have the benefit that they may be altered to incorporate automatic kinematic and kinetic data collection allowing the possibility of quantitative measures of subject performance and recovery of function. While there are many devices reported at present, the common feature is their facilitation of repetitious exercise. The most notable recent developments which provide context for this work are electromyogram feature-triggered systems reported by Dipietro [30]. One of the advantages highlighted by Dipietro and her colleagues is that “ *It may allow highly-impaired subjects to activate robot assistance; such patients might be able to generate EMG signals even though they were unable to produce sufficient movement to trigger the robot* .” Previous systems rely on exceeding kinematic/kinetic thresholds based on limb velocity, for example, to trigger movement. Therefore, Dipietro's system can be regarded as harnessing peripheral nervous system activity as recorded through electrical muscular activity to trigger feedback. It is proposed in the present paper that central nervous system activity measures may serve as an alternative trigger—a concept that suggests a new application area for brain-computer interfaces.

### 2.2. Brain-computer interfaces in neurorehabilitation

Brain-computer interfaces (BCIs) are devices that act as neural prostheses. They facilitate communication or information transfer between the brain and the outside world independent of the peripheral nervous system. While the primary focus of BCI research within neurorehabilitation has been to provide assistive technology to enable communication for the severely disabled, there have been suggestions that the technology may have something to offer in terms of physical recovery for certain conditions through reinforcement of damaged neural pathways [31], plasticity-induced cortical reorganization [32], and triggering of functional electrical stimulation [33]. While there has been movement of BCI research towards this area, most rehabilitation efforts have been directed towards harnessing neural prostheses for controlling robotic limbs for reaching and manipulating tasks or control of wheelchairs. To these particular ends, great progress has been made in terms of practicality [34, 35], speed [36, 37], and ease of use [38]. Such advances are continuing, however the more subtle application of the technology as a biofeedback mechanism for physical rehabilitation has hitherto been underdeveloped. One of the most impressive attempts in this direction is the Brain-Orthosis-Interface reported as a solution for chronic stroke sufferers [39]. The technology based on magnetoencephalography methods monitors sensorimotor rhythm to derive control signals to open and close an orthotic hand coupled to the patient's own. In this way, the patient receives enhanced feedback through both watching and feeling their own hand moving. Such operant conditioning enhances the biofeedback process and improves neural prosthesis performance. Such a case represents a more extreme rehabilitative application of a BCI in that the neural prosthesis is a permanent one. The paradigm presented in this paper casts the BCI in the role of a temporary neural prosthetic splint that provides feedback in lieu of feedback from actual movement. The contribution of this paper is in this context. When, if ever, movement, however minimal, is restored, more conventional forms of CIMT may be applied probably removing the necessity for the BCI. A related concept is the provision of an afferent neural prosthetic for rehabilitation. In a recent work, transcranial direct current stimulation of the motor cortex is used to improve rehabilitation outcome [40]. This can be interpreted as a neural prosthetic encouraging cortical activation associated with movement.

### 2.3. Near infrared spectroscopy-based brain-computer interface

A near infrared spectroscopy-based brain-computer interface utilises an optical modality for inferring changes in brain state. It is possible to measure changes in cerebral blood volume and oxygenation associated with cortical activity through the use of light in the 600—1000 nm wavelength range yielding a cerebral haemodynamic monitor. The optical absorption and scattering properties of scalp, hair, skull, and the meninges surrounding the brain allow photons of these wavelengths to penetrate in to the surface of the cortex where they undergo scattering and absorption events with a wide range of chromophores in the tissue. The significant scattering component means that a small proportion of the injected light will exit at some distance from the source carrying information about chromophore concentration dynamics at the upper surface of the brain. A suitably sensitive well-positioned detector can detect this photon flux and allow noninvasive monitoring of these changes. There are a number of chromophores in brain tissue in this band whose optical properties are correlated with mental activation. Of these, the most germane is haemoglobin—the oxygen carrying molecule of the body. Haemoglobin exists principally in two forms in the body: an oxidised state and a reduced state. These two states generally referred to as oxyhaemoglobin (HbO) and deoxyhaemoglobin (Hb) have distinctly different absorption spectra allowing their relative concentrations to be determined through multiple wavelength interrogation. During concerted cortical activity, a neurovascular process occurs whereby changes occur in cerebral blood flow, volume, and metabolic rate of consumption. This manifests itself principally as an increased demand for oxygen with the local vasculature responding through flooding the cortical area and surrounding tissue with oxygenated haemoglobin. Usually this is accompanied by a corresponding drop in deoxyhaemoglobin concentration—a component thought to be responsible for the signal recorded during fMRI. The relative changes in haemoglobin can be distinguished through interrogation at a number of wavelengths in the near infrared band described above and therefore changes in cortical activation associated with mental activity can be monitored. This is the basis of NIRS-BCI. A detailed review of near infrared spectroscopy techniques for biomedical application can be found in [41].

The measurement principle in more quantitative terms can be expressed using a modified version of the Beer-Lambert Law. The attenuation due to absorption and scattering effects may be described therefore as

(1) $$A=\log_{10\,}\,\frac{I_{0}}{I}=\alpha cLB+G$$

Here *A *is attenuation, *I_0_* is incident light intensity, *I* is transmitted light intensity. On the right-hand side, αis the specific extinction coefficient for the absorber which is wavelength dependent in this case, *c* is the concentration of the absorber, *L* is the distance between the source and detector, *B* is the differential path length factor, and *G* is a term to account for scattering losses.

Changes in haemoglobin levels are calculated then as a superposition of the changes for each absorber species—in this case oxy- and deoxyhaemoglobin:

(2) $$\Delta A=\left(\alpha_{\text{HbO}}\Delta_{c\text{HbO}}+\alpha_{\text{Hb}}\Delta_{c\text{Hb}}\right)BL$$.

Equation (2) is evaluated at two wavelengths, either side of the isobestic point to enable separation of the two haemoglobin states.

Previous functional NIRS studies have documented haemodynamic changes as a result of motor, cognitive, visual, and auditory activities [41]. The device used here has been used previously to record evoked responses arising from motor imagery in the sensorimotor cortex [19]. The general form is an increase in HbO coupled with a decrease in Hb 3–5 seconds after the onset of movement execution or imagery. While the idea of monitoring cerebral oxygenation using NIRS has been around for some time, it has as yet found only limited application in brain-computer interfacing mostly due to the slow baud rate of the device. Currently, two working devices have been reported [17, 18], however the area is nascent and undoubtedly more in-depth investigations of the efficacy of such devices will appear.

As an illustration of how CIMT might be augmented through the provision of biofeedback of motor cortical effort, the next section describes a simple practical embodiment in which a NIRS-BCI is used to trigger movement of a robotic actuator as a result of elevated motor cortical activity.

## 3. AN ILLUSTRATIVE EMBODIMENT

An example of how an embodiment of the concept described in this paper might work is now given based on a synchronous BCI paradigm in which the activation signal is derived from bilateral cortical activity over the sensorimotor region (SMR). Unlike most BCI experiments, however, overt motor activity is employed by the subjects as imagined activity is not required or indeed germane for the rehabilitative therapy envisaged. Actual movement allows the experimenter to determine that the motor areas must indeed be active and hence eliminates the effect of poor engagement on the part of the subjects in the results. A computer is used to present movement instructions to the subjects (audio and visual cues). Appropriate activation of the SMR during movement triggers feedback in the form of movement of a robotic arm.

The signals, collected simultaneously, are cerebral changes in HbO and Hb, the respiration pneumogram and the digital photoplethysmograph (PPG).

### 3.1. Hardware

A continuous wave dual channel NIRS system is used to interrogate the cerebral cortex on each hemisphere. The light source comprises light emitting diodes (LEDs) at wavelengths of 760 nm and 880 nm (Opto Diode corp., Inc., APT-0010/OD-880F, Calif, USA) having a narrow beam angle of and a spectral bandwidth at 50% of 30 nm and 80 nm, respectively. The light output of each LED is modulated in the low kilohertz range to facilitate lock-in detection at the output. The LEDs are placed in direct contact with the scalp. Avalanche photodiodes (APD), Hamamatsu C5460-01, were used as the detector; the output of which was fed via a 3 mm diameter, 1 m long, fibre optic bundle to lock-in amplifiers (Signal Recovery, model 7265). A more detailed account of the optical setup and other design considerations can be found in [42].

For data acquisition (offline analysis), the *Biopac* *UIM100C interface module* in tandem with a *Biopac* *MP100* was used to collect the four analogue channels of NIRS data (two wavelengths, two sites) from the lock-in amplifiers at 16-bit resolution. In addition, two other analogue channels of data were collected by the *MP100* for respiration and PPG (*Biopac* amplifiers models *PPG100C* and *RSP100C*) with gains of 100 and 10, respectively. *PPG100C* settings comprised a low-pass filter of 10 Hz and high-pass filter of 0.05 Hz. *RSP100C* settings implemented a low-pass filter of 10 Hz.

**Figure 1:**
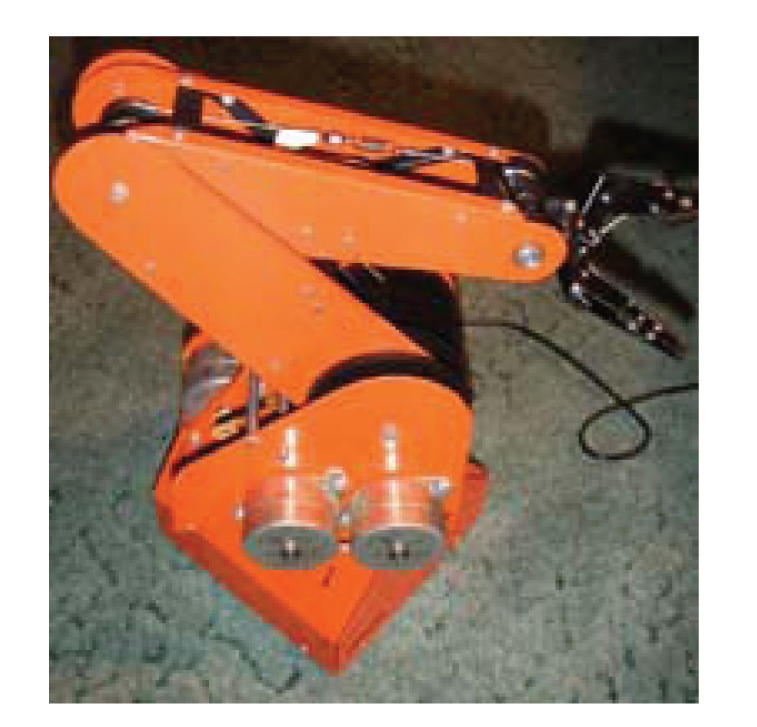
The Armdroid-1 robotic arm used in the feedback protocol.

Feedback was provided through movement of a robotic arm (Figure 1) in sympathy with sustained elevation in HbO levels during the motor execution tasks. The outputs of the lock-in amplifiers was tapped to provide drive signals via a simple *12-bit National Instruments USB-6008* DAQ at 10 samples per second. Online and real-time processings for Hb and HbO using standard algorithms [43] based on (2) provided control of the robotic arm and feedback. The robotic arm was driven using control signals from the DAQ system allowing predetermined movement patterns to be invoked when haemodynamic activity exceeded rest period levels.

### 3.2. A simple experimental protocol

In this work, we enlisted 5 healthy subjects (4 males, 1 female), 2 left handed and 3 right handed (determined, using Edinburgh Handedness Inventory [44]). The subjects age range was 23-25 years old (mean age 24years). One subject (female) was removed from analysis due to poor SNR and optode placement problems. The remaining four subjects underwent online feedback experiments. All remaining subjects were included in the full analysis. Two of the subjects had no previous experience with NIRS experiments.

**Figure 2:**
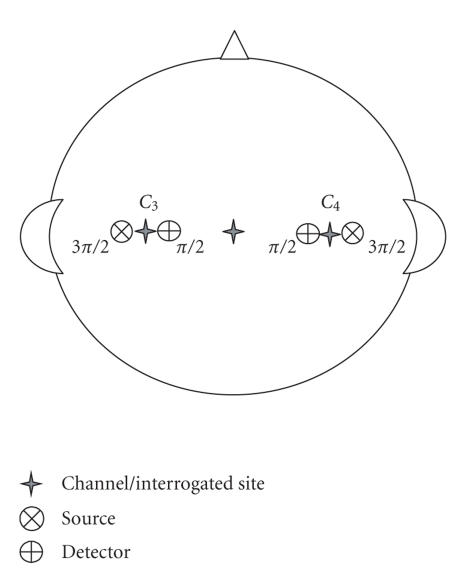
Illustration of relative positioning of optode sources and detectors.

Each subject was seated in a near supine position to reduce the effects of low-frequency blood oscillations (Mayer wave) in a dimly lit room. The respiration monitoring device (*Biopac-TSD201*) was strapped around the chest of each subject to monitor the respiratory signal during trials. The PPG probe (*Biopac-TSD200*) was attached to the index finger on the inactive limb to monitor the cardiac pulse during trials. Subjects' head measurements were taken to locate positions and . These 10–20 system positions are approximately over primary motor cortex centres in the brain responsible for right- and left-hand movements. The distance between the source and detector was 30 mm. A more precise positioning descriptor is available using the optode placement system proposed in [45]. Using this system, the optode location is described in terms of distance and angle with respect to a defined EEG 10–20 system landmark position which serves as an origin. In this study, angles are referenced to *C_z_*. This yields optode descriptors as in Table 1, illustrated in Figure 2.

Hair was parted under the optode for both the source and detector to leave ample hair-free scalp. The optodes and fibre optic bundles were inserted into cushioned pads in contact with the subject's scalp. The subject's hands were placed under restraining straps in order to facilitate isometric exercise during the stimulus trials. Once positioned and instrumented, the subject was given instructions to follow, before commencing the experiment. Prior to experiment each subject was informed about the nature and purpose of the experimental study and given precise instruction as to the task required of them. To reduce artefact ,subjects were asked to minimise head and body movements as well as given instructions to breathe gently and regularly.

**Figure 3:**
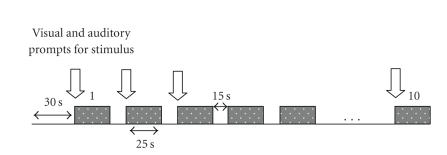
Illustration of the experimental sequencing. Shaded boxes are motor task periods.

The paradigm for performing the overt motor task is shown in Figure 3. An initial 30 seconds rest was followed by alternating periods of 25 seconds of motor effort (isometric maximal voluntary contractions —MVCs of the indicated forearm, pivoting at the elbow on a rigid support surface) and 15-second rest. For each “experimental session,” there were 10 stimulus periods. Each of the four subjects carried out two sessions on each arm, thus a total of 20 stimulus periods per arm per subject. Thus, a total of 80 online trials for each left and right arm are used in the final analysis.

Audio-visual cues indicating the task and rest periods were presented via an LCD monitor to the subjects. Feedback was provided in two forms: a symbolic form which on the LCD monitor presented itself as a change from a black rectangle to an upwards pointing arrow when HbO levels in excess of the previous rest period's level were present, and a physical action cue where movement of the robotic arm took place under the same conditions. When the HbO levels dipped below the threshold during the motor task period, the icon reverted to the black rectangle form and motion of the robotic arm ceased.

### 3.3. A basic signal classification scheme

Raw signals from the lock-in amplifiers were sampled at 10 Hz, and the Hb and HbO concentrations were calculated in real-time, on a sample-by-sample basis. Simple moving average filters were used in all experiments. A 10-point moving average filter was used to low-pass filter data in real time. Once Hb and HbO concentrations were calculated, a further moving average filter was used for classification. For the detection of significant activity during the activation period, a simple thresholding scheme was employed whereby a datum was taken during the preceding rest period. This datum consisted of the average HbO level during the 15 seconds of the rest period. Neither Hb nor total haemoglobin levels were used as an information signal in the online experiments. The 10 point running average of the HbO signal calculated during the motor task period was thresholded against this reference signal. When the level was exceeded during this period, significant motor cortical activity was inferred and appropriate feedback was presented. In summary, activation occurs where $s[i]-\overset{¯}{r}>0$,

(3) $$s\left[i\right]\;=\frac{1}{W}\,\overset{W-1}{\underset{J=1}{\sum }}\;\text{HbO}\left[i+j\right]\;\text{for}\;i\,\;=1,\ldots ,N,$$

where *s*[*i*] is the derived control signal at the *i*th sample, *W* = 10, *N* is the number of samples acquired during the motor task and $\overset{¯}{r}$is the average HbO signal during the rest period.

So long as the stimulus moving average was greater than the rest average, activity was sensed and the robotic arm was activated.

### 3.4. Results

**Figure 4:**
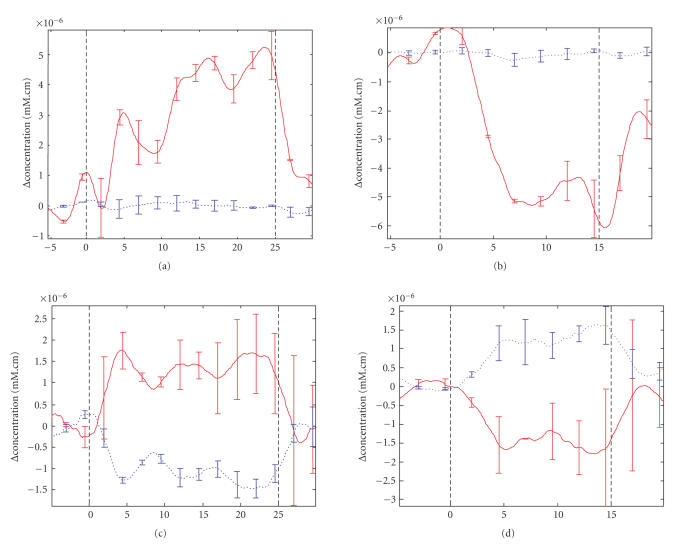
Top row shows average Hb (dashed trace) and HbO (solid trace) levels SD for subject 2. The bottom row shows average readings for subject 1. The left-hand column shows activity during motor task (between vertical dashed lines) while the right-hand column shows corresponding activity during rest. The abscissa for all plots is in seconds.

Table 2 presents the results of the experiment as described. This table shows the percentage of time that subjects were able to move the robot during the motor activation task. All the subjects were successful in achieving some control of the robotic arm. For example, subject 1 was able to activate the robot almost all the time when engaged in right forearm movement (>95%). Subject 3 unfortunately was not as successful as the others, only realising movement of the robot arm just over 60% of the time (a footnote to the table may suggest why). However, the measures presented here are rather conservative as they indicate the percentage of time by which the threshold was exceeded during the motor task. If the results were reworked to indicate the percentage of motor task periods where the robotic arm was activated, then the results would be almost perfect. This of course would be a disingenuous summary of the experiment for many reasons. A more insightful observation of the experiments can be obtained from Figure 4 which shows the averaged responses (including standard deviations) for two paradigmatic subject tests during both the motor task and rest periods.

Figure 4 was produced using the Matlab-based NIRS analysis tool HomER [46] and illustrates mean and standard deviation levels that indicate consistent differences between rest and activation. The smoothness of the plot is attributable both to averaging over all trials from both channels and-a 3rd to order low-pass Butterworth filter with cutoff frequency of 0.7 Hz implemented before calculating averages and standard deviations.

## 4. POTENTIALS AND PROSPECTS

The key contribution of this paper is the presentation of the idea that a neurocortical prosthesis may serve as a means to extend CIMT to severe stroke sufferers as part of an therapeutic regime. The very simple illustration of this idea in Section 3 highlights well the basic operation of a NIRS-BCI in a CIMT-like scenario. It is reasonable to suggest that even the toy system above may provide a basic platform on which to develop more0 sophisticated systems for comprehensive studies with the intended population of stroke sufferers. The results, which in themselves are nonsurprising in nature, are useful for facilitating assessment of potential design issues for more developed systems with the caveat that acquiring good quality signals may be difficult with damaged cortex and that even with robust signals, there is perhaps the possibility of habituation effects which may limit applicability. Notwithstanding these concerns, the responses and activation levels evident in Figure 4 show all the characteristics expected [18, 42] of NIRS-BCI signals. While the results show high variability, they have been calculated for real-time biofeedback. This presents a significantly more difficult scenario than offline analysis which would allow for removal of artefact and screening of signals and would undoubtedly show improved figures. However, real-time control is important for the application envisaged and the results do indicate that such biofeedback is possible. The signal processing necessary crude, given the real-time requirements, is certainly worth more sustained development to compensate for motion-contaminated trials and other extrageneous forms of artefact. Online adaptive filtering is a necessary component for a more robust system. Clearly, a better understanding of the underlying responses may allow better integration of other signals such as the Hb signal. The variability of the Hb response meant that it was difficult to reliably use it as a trigger signal and although discarded here, it is a useful signal to collect for future work in improving performance. The idea of integrating other signals can be taken further, for example, the provision of a multimodal neurocortical prothesis harnessing motor rhythm EEG would clearly enhance the system further as it is probable that the compound signal would offer greater sensitivity to weaker cortical activation and better insight into neurological function [47]. In addition, such a system could provide some measures of neurovascular coupling; the parameterisation of which may lead to greater insight in the rehabilitative process. It is also worth considering if perhaps a motor-rhythm EEG BCI may work better as a neurocortical prosthesis for these applications independent of any vascular response-oriented method. Only further research will answer this.

The work reported in this paper clearly represents only first steps towards extending CIMT to more severe motor stroke patients and the authors would be first to admit that there are very many questions unanswered which all merit further exploration. One obvious question from the technological point of view taken here is whether or not the haemodynamic signal required is as pronounced for sufferers of stroke. In the case of cortical haemorrhagic stroke, for example, the presence of scar tissue and haemotoma may absorb a significant portion of the introduced near infrared light attenuating the signal. Similarly constructed arguments may be made for ischemic stroke; however in all cases, the severity of such effects if they occur at all clearly depends on the site and extent of the injury. One might envisage that initial fMRI scans during attempted movement would facilitate the deployment of the optode configurations required in such cases. To answer these concerns, clinical trials are required with appropriately selected stroke patients. An intriguing possibility for controlled studies is the monitoring of changes in the haemodynamic signals themselves along with motor movement efficacy. Additional quantitative measures such as those which might be provided through this method would surely prove useful in measuring rehabilitative outcome. The pioneers in this area have noted in a recent paper that NIRS monitoring may provide a technological breakthrough in terms of developing and understanding CIMT [9]. Techniques such as the one espoused here may make some contribution to the realisation of this suggestion. There are other concerns too. As mentioned during the background section, the neural prosthesis advocated here is not intended as a permanent replacement for the patient's own nervous system. It is envisaged that the device serve as a temporary channel to convey some feedback for stroke sufferers who have none. As soon as any other more conventional feedback is available, then the prosthesis may be discarded. This philosophy is suggested in response to suggestions that repetitive use of stereotyped brain signals metabolic in origin or otherwise could within this disease context produce unwanted plasticity phenomena such as tics, obsessive thoughts, and other aberrant neurological functioning [32].

## 5. SUMMARY

This paper has highlighted the possibility of enhancing the application of CIMT for stroke sufferers through the addition of a neuro-cortical prosthesis. Generally it is proposed that a fruitful avenue for new research in the application of brain computer interfaces is in their measurement of volitional motor effort for biofeedback purposes in CIMT. The intended target treatment group is severe stroke patients with little or no perceptible movement although the idea may have utility in the broader stroke population. More specifically NIRS-based BCI are proposed as suitable candidates for such purposes. A simplified illustration of such a system is presented which demonstrates the basic feasibility of the approach. Testing with actual stroke sufferers is clearly the next step and will in itself undoubtedly introduce a number of significant challenges. Nevertheless we believe the concept described in this paper has merit as a specific extension of brain computer interfaces into the neurorehabilitation domain.

## Figures and Tables

**Table 1:** Optode locations referenced to EEG 10–20 system.

Optode descriptions	Light source location	Detector location
Channel 1 (left-hand side)	*C*3 : 1.5(3π / 2)	*C*3 : 1.5(π / 2)
Channel 1 (right-hand side)	*C*4 : 1.5(3π / 2)	*C*4 : 1.5(π / 2)

**Table 2:** Success rate in moving robot arm. Figures indicate the percentage of time subjects were able to keep the robot moving during each trial. That is, subject 4 successfully moved the robot 96.7% of the time during all 10 stimulus trials for the first session of left-arm maximum voluntary contraction (left 1).

Subject	Left 1 (%)	Left 2 (%)	Right 1 (%)	Right 2 (%)	Subject average (%)
1	87.2±23.9	90.4±11.0	91.8±15.9	95.4±5.1	91.2±13.9
2	82.4±20.2	88.2±13.4	73.5±25.9	82±25.2	81.5±21.2
3*	74.7±25.8	64.3±21.7	63.7±33.6	46.8±38.7	62.4±29.9
4	96.7±3.2	98.4±2.9	90.2±19.1	86.5±23.6	93±12.2

*Subject 3 experiments had low-light levels, thus a lower SNR. A previous X-ray has also shown that he has a relatively thick skull.

## References

[1] Adams R, Victor M, Ropper A (1997). *Adam's & Victor's Principles of Neurology*.

[2] World Health Organization Global Report 2002 (2007). World Health Organization. http://www.who.int/whr/2002/en/index.html.

[3] The Internet Stroke Centre (2007). http://www.strokecenter.org/pat/stats.htm.

[4] Bohannon RW (2007). Muscle strength and muscle training after stroke. *Journal of Rehabilitation Medicine*.

[5] Taub E, Miller NE, Novack TA (1993). Technique to improve chronic motor deficit after stroke. *Archives of Physical Medicine and Rehabilitation*.

[6] Taub E, Uswatte G, Pidikiti R (1999). Constraint-induced movement therapy: a new family of techniques with broad application to physical rehabilitation—a clinical review. *Journal of Rehabilitation Research and Development*.

[7] Miltner WH, Bauder H, Sommer M, Dettmers C, Taub E (1999). Effects of constraint-induced movement therapy on patients with chronic motor deficits after stroke: a replication. *Stroke*.

[8] Liepert J (2006). Motor cortex excitability in stroke before and after constraint-induced movement therapy. *Cognitive and Behavioral Neurology*.

[9] Mark VW, Taub E, Morris DM (2006). Neuroplasticity and constraint-induced movement therapy. *Europa Medicophysica*.

[10] Wolf SL, Winstein CJ, Miller JP (2006). Effect of constraint-induced movement therapy on upper extremity function 3 to 9 months after stroke: the EXCITE randomized clinical trial. *Journal of the American Medical Association*.

[11] Mark VW, Taub E (2004). Constraint-induced movement therapy for chronic stroke hemiparesis and other disabilities. *Restorative Neurology and Neuroscience*.

[12] Lum PS, Burgar CG, Kenney DE, Van der Loos HFM (1999). Quantification of force abnormalities during passive and active-assisted upper-limb reaching movements in post-stroke hemiparesis. *IEEE Transactions on Biomedical Engineering*.

[13] van der Lee JH, Wagenaar RC, Lankhorst GJ, Vogelaar TW, Devillé WL, Bouter LM (1999). Forced use of the upper extremity in chronic stroke patients: results from a single-blind randomized clinical trial. *Stroke*.

[14] Chae J, Bethoux F, Bohinc T, Dobos L, Davis T, Friedl A (1998). Neuromuscular stimulation for upper extremity motor and functional recovery in acute hemiplegia. *Stroke*.

[15] Powell J, Pandyan AD, Granat M, Cameron M, Stott DJ (1999). Electrical stimulation of wrist extensors in poststroke hemiplegia. *Stroke*.

[16] Butler AJ, Page SJ (2006). Mental practice with motor imagery: evidence for motor recovery and cortical reorganization after stroke. *Archives of Physical Medicine and Rehabilitation*.

[17] Coyle S, Ward TE, Markham C, McDarby G (2004). On the suitability of near-infrared (NIR) systems for next-generation brain-computer interfaces. *Physiological Measurement*.

[18] Sitaram R, Zhang H, Guan C (2007). Temporal classification of multichannel near-infrared spectroscopy signals of motor imagery for developing a brain-computer interface. *NeuroImage*.

[19] Coyle S, Ward TE, Markham C (2004). An optical brain computer interface.

[20] Krakauer JW (2006). Motor learning: its relevance to stroke recovery and neurorehabilitation. *Current Opinion in Neurology*.

[21] Taub E, Uswatte G (2006). Constraint-induced movement therapy: answers and questions after two decades of research. *NeuroRehabilitation*.

[22] Krebs HI, Hogan N, Aisen ML, Volpe BT (1998). Robot-aided neuro-rehabilitation. *IEEE Transactions on Rehabilitation Engineering*.

[23] Plautz EJ, Milliken GW, Nudo RJ (2000). Effects of repetitive motor training on movement representations in adult squirrel monkeys: role of use versus learning. *Neurobiology of Learning and Memory*.

[24] van der Lee JH, Wagenaar RC, Lankhorst GJ, Vogelaar TW, Devillé WL, Bouter LM (1999). Forced use of the upper extremity in chronic stroke patients: results from a single-blind randomized clinical trial. *Stroke*.

[25] Volpe BT, Krebs HI, Hogan N, Edelsteinn L, Diels CM, Aisen ML (1999). Robot training enhanced motor outcome in patients with stroke maintained over 3 years. *Neurology*.

[26] Ferraro M, Palazzolo JJ, Krol J, Krebs HI, Hogan N, Volpe BT (2003). Robot-aided sensorimotor arm training improves outcome in patients with chronic stroke. *Neurology*.

[27] Colombo R, Pisano F, Micera S (2005). Robotic techniques for upper limb evaluation and rehabilitation of stroke patients. *IEEE Transactions on Neural Systems and Rehabilitation Engineering*.

[28] Volpe BT, Ferraro M, Lynch D (2005). Robotics and other devices in the treatment of patients recovering from stroke. *Current Neurology and Neuroscience Reports*.

[29] Johnson MJ (2006). Recent trends in robot-assisted therapy environments to improve real-life functional performance after stroke. *Journal of NeuroEngineering and Rehabilitation*.

[30] Dipietro L, Ferraro M, Palazzolo JJ, Krebs HI, Volpe BT, Hogan N (2005). Customized interactive robotic treatment for stroke: EMG-triggered therapy. *IEEE Transactions on Neural Systems and Rehabilitation Engineering*.

[31] Kübler A, Mushahwar VK, Hochberg LR, Donoghue JP (2006). BCI meeting 2005-workshop on clinical issues and applications. *IEEE Transactions on Neural Systems and Rehabilitation Engineering*.

[32] Dobkin BH (2007). Brain-computer interface technology as a tool to augment plasticity and outcomes for neurological rehabilitation. *The Journal of Physiology*.

[33] Pfurtscheller G, Müller GR, Pfurtscheller J, Gerner HJ, Rupp R (2003). ‘Thought’—control of functional electrical stimulation to restore hand grasp in a patient with tetraplegia. *Neuroscience Letters*.

[34] Buttfield A, Ferrez PW, Millán JDR (2006). Towards a robust BCI: error potentials and online learning. *IEEE Transactions on Neural Systems and Rehabilitation Engineering*.

[35] Wang Y, Wang R, Gao X, Hong B, Gao S (2006). A practical VEP-based brain-computer interface. *IEEE Transactions on Neural Systems and Rehabilitation Engineering*.

[36] McFarland DJ, Krusienski DJ, Wolpaw JR (2006). Brain-computer interface signal processing at the Wadsworth Center: mu and sensorimotor beta rhythms. *Progress in Brain Research*.

[37] Sellers EW, Krusienski DJ, McFarland DJ, Vaughan TM, Wolpaw JR (2006). A P300 event-related potential brain-computer interface (BCI): the effects of matrix size and inter stimulus interval on performance. *Biological Psychology*.

[38] Vidaurre C, Schlögl A, Cabeza R, Scherer R, Pfurtscheller G (2006). A fully on-line adaptive BCI. *IEEE Transactions on Biomedical Engineering*.

[39] Birbaumer N, Cohen LG (2007). Brain-computer interfaces: communication and restoration of movement in paralysis. *The Journal of Physiology*.

[40] Hummel F, Celnik P, Giraux P (2005). Effects of non-invasive cortical stimulation on skilled motor function in chronic stroke. *Brain*.

[41] Rolfe P (2000). In vivo near-infrared spectroscopy. *Annual Review of Biomedical Engineering*.

[42] Coyle S (2005). *Near-infrared spectroscopy for brain computer interfacing, Ph.D. thesis*.

[43] Cope M, Delpy DT (1988). System for long-term measurement of cerebral blood and tissue oxygenation on newborn infants by near infra-red transillumination. *Medical and Biological Engineering and Computing*.

[44] Oldfield RC (1971). The assessment and analysis of handedness: the Edinburgh inventory. *Neuropsychologia*.

[45] Coyle S, Markham C, Lanigan W, Ward T A mechanical mounting system for functional near-infrared spectroscopy brain imaging studies.

[46] HomER http://www.nmr.mgh.harvard.edu/PMI/resources/homer/home.htm.

[47] Babiloni F, Mattia D, Babiloni C (2004). Multimodal integration of EEG, MEG and fMRI data for the solution of the neuroimage puzzle. *Magnetic Resonance Imaging*.

